# Shunt dysfunction patterns after transjugular intrahepatic portosystemic shunt creation using a combination of a generic stent-graft and bare-stents

**DOI:** 10.1186/s42155-023-00421-7

**Published:** 2024-01-10

**Authors:** Guillaume Gravel, Florent Artru, Miriam Gonzalez-Quevedo, Georgia Tsoumakidou, Nicolas Villard, Rafael Duran, Alban Denys

**Affiliations:** 1 Department of Diagnostic and Interventional Radiology, Lausanne University Hospital and University of Lausanne, Lausanne, Switzerland; 2 Department of Liver Diseases, Rennes University Hospital, Rennes, France; 3 Institute of Liver Studies, King’s College Hospital, London, UK; 4 Department of Gastroenterology and Hepatology, Lausanne University Hospital, Lausanne, Switzerland

**Keywords:** Transjugular intrahepatic portosystemic shunt, TIPS or Stent Dysfunction, Angiography, Stenosis

## Abstract

**Purpose:**

Even though transjugular intrahepatic portosystemic shunt (TIPS) using Fluency Stent-grafts provides good shunt patency rates, shunt dysfunction is a great concern after TIPS creation, occurring in up to 20% of cases within one year. The objective of this study was to describe shunt dysfunction patterns after TIPS creation using a combination of generic stent-grafts/bare-stents.

**Materials and methods:**

Single-center retrospective study of all TIPS revisions between January 2005 and December 2020. TIPS revision angiograms were analyzed for stents’ positions, stenoses’ diameters, and stenoses’ locations.

**Results:**

Out of 99 TIPS, a total of 33 TIPS revisions were included. The median time to TIPS revision was 10.4 months. Angiograms showed four patterns of TIPS dysfunction-associated features (DAF), defined as follows: Type 1 was defined as stenosis located after the stent end in the hepatic vein (HV), type 2 as intra-stent stenosis located in the hepatic vein, type 3 as intra-stent stenosis or a kink in the parenchymal tract or the portal vein end of the TIPS, and type 4 as a complete TIPS occlusion. Types 1, 2, 3, and 4 were seen in 23 (69.7%), 5 (15.2%), 2 (6.1%), and 3 (9.1%) TIPS respectively. TIPS revision was successful in 30 (90.1%) patients with median pre- and post-TIPS revision PSG of 18.5 mmHg and 8 mmHg respectively (*p* < .001).

**Conclusion:**

Our results illustrate the four angiographic patterns of TIPS DAF after TIPS creation using a combination of generic stent-grafts/bare-stents and emphasize the need for appropriate stent length extending to the HV/inferior vena cava junction.

## Introduction

Transjugular intrahepatic portosystemic shunt (TIPS), first described by Rösch et al. in 1969 [1], is an effective tool for the treatment of variceal bleeding, refractory ascites, refractory hepatic hydrothorax secondary to liver cirrhosis and Budd-Chiari syndrome [2–4]. Covered stents for the creation of TIPS are now recommended as they confer a higher and longer patency rate when compared to bare stents [2–4]. Many interventional radiologists use the Viatorr stent-graft (W. L. Gore and Associates, Newark, DE, USA) introduced in 1999 as the first dedicated TIPS stent-graft. The Viatorr is a nitinol self-expendable stent-graft with an uncovered segment of 2 cm in its proximal end designed to extend into the portal vein. The ideal location of the distal end of the stent-graft is at the junction of the hepatic vein (HV) with the inferior vena (IVC) [5, 6]. However, the covered portion of the Viatorr stent-graft extending to the HV-IVC junction risks the occlusion of the selected HV, with HV thrombosis occurring in approximately 16% of patients after TIPS creation using Viatorr stent-grafts [7]. Occlusion of the selected HV may increase the risk of liver ischemia after TIPS creation [8, 9] and cause liver failure and abscess formation [10, 11]. One of the alternatives to the Viatorr is the Fluency stent-graft which has shown good shunt patency rates [12–19]. Since 2005, TIPS have been performed in our institution using a generic stent-graft (Fluency stent-graft, Becton Dickinson, Franklin Lakes, NJ, USA) covering the hepatic track length in association with bare self-expanding stents extending in the portal vein and to the junction of the HV with the IVC. This technique has a theoretical advantage over the use of the Viatorr stent-graft with the possibility to maintain the selected HV permeable, the reason why we use the combination of a generic stent-graft with one or more self-expendable bare stents. However, this advantage might be balanced by a higher risk of shunt dysfunction which commonly occurs within the first 12 months in 7 to 19% of cases [12–19]. We wondered whether stent dysfunctions observed after shunt creation using a combination of a generic stent-graft and bare-stents were related to a particular occlusion site, that could potentially be prevented during the procedure. The objective of this study was therefore to describe the shunt dysfunction patterns of TIPS created using a combination of a generic stent-graft and bare-stents.

## Materials and methods

### Data collection

We have retrospectively included all patients who underwent TIPS revision in our institution from January 2005 to December 2020. Institutional review board approval was obtained. Patients’ records were reviewed for patients’ demographics, disease information, Child–Pugh score, MELD (Model for End-stage Liver Disease) score, procedures, and clinical outcomes. TIPS procedures and revisions procedures were analyzed for stenoses’ diameters, stenoses’ locations, stents’ diameters, stents’ positions, and dilatation diameter.

**Fig. 1:**
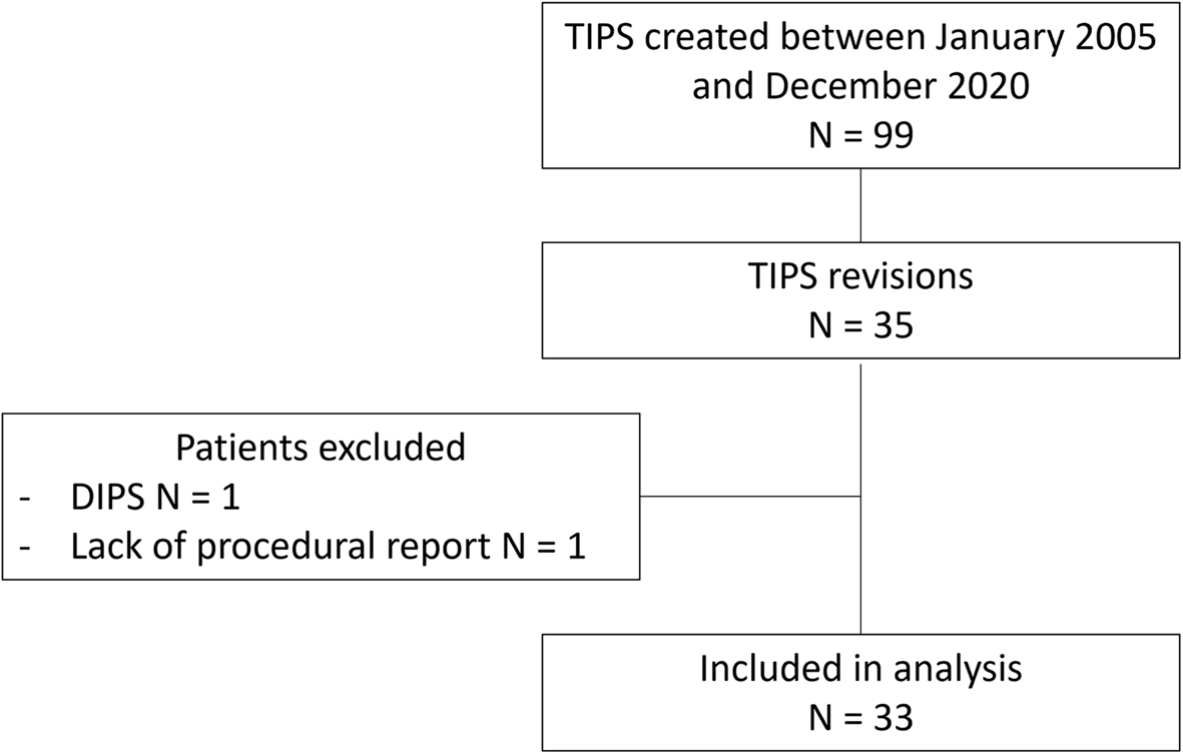
Flow Chart

Exclusion criteria were (1) direct intrahepatic portocaval shunt (DIPS) creation; (2) no retrievable medical records (Fig. 1).

### TIPS procedure

All procedures were performed by a senior interventional radiologist under general anesthesia using ultrasound and fluoroscopy guidance. The pre-TIPS portal venous pressure was obtained before hepatic parenchymal tract dilatation and the post-TIPS portal venous pressure was obtained after stent deployment and dilatation. All patients underwent hepatic parenchymal tract dilatation before stent placement with a balloon catheter of 4 or 6 mm in diameter. One self-expendable 8–12 mm × 40–80 mm Fluency stent-graft was inserted to cover the length of the hepatic parenchymal tract. One or two bare stents of 10–12 mm × 40–100 mm (Venovo, Becton Dickinson, Franklin Lakes, NJ, USA) were inserted coaxially within the stent-graft to extend the shunt to the HV/IVC junction distally and into the portal vein proximally. After stent insertion, the hepatic parenchymal tract was dilated with a 6-, 8- or 10-mm balloon catheter depending on the post-TIPS portosystemic gradient (PSG).

### Follow-up evaluation

The follow-up doppler US evaluation to determine the TIPS patency used in our center was the following: first doppler US within 2 weeks, then every 3 to 6 months or as clinically indicated.

### TIPS revision

TIPS dysfunction was suspected in case of recurrent symptoms of portal hypertension or if follow-up ultrasound showed peak intra-shunt velocity ≥ 250 cm/sec, maximum velocity in the portal third of the shunt ≤ 50 cm/sec, or maximum portal vein velocity less than or equal to two-thirds of the baseline value [20]. Patients were then addressed for TIPS revision with pre-revision PSG measurement and an angiogram of the portal vein was performed. TIPS revision procedures were performed by a senior interventional radiologist under local anesthesia or general anesthesia depending on the patient’s clinical condition. Angioplasty and/or new stent insertion were performed if a stenosis > 50%, a kink (i.e. angulation > 90°), or a thrombosis was highlighted at the angiogram, or if the pre-TIPS revision PSG was above 12 mmHg. TIPS revision procedure was considered successful if TIPS was recanalized with no residual significant stenosis (> 50% or kink) or if the post-TIPS PSG was below 12 mmHg.

### Statistical analysis

Statistical analysis was performed using EZR software [21]. Data were expressed as the median (range) and as counts and percentages when appropriate. Comparison between pre- and post-TIPS creation and revision PSG was performed using the paired t-test. A *P* value ≤ 0.05 was considered significant.

## Results

### Patients

A total of 99 patients had a TIPS creation in our institution between January 2005 and December 2020. Of those patients, 33 (24M:9F, age range 44–71 years) met the selection criteria. The median MELD and Child–Pugh scores at the time of the TIPS creation were 11 (range 6–26) and 8 (range 5–14) respectively. Etiologies of cirrhosis were alcohol-associated liver disease in 20 (60.6%) patients, mixed in 5 (15.2%) patients, nonalcohol-associated fatty liver disease (NAFLD) in 4 (12.1%) patients, chronic viral hepatitis in 3 (9.1%) patients, and hemochromatosis in 1 (3.0%) patient.

### TIPS procedures

Indications for TIPS creation were variceal hemorrhage (salvage or preemptive TIPS) in 16 patients (48.5%), refractory ascites in 14 patients (42.4%), and prophylactic placement before abdominal surgery in 3 patients (9.1%). The median stent-graft diameter was 10 mm (range 8–12) with a median tract dilatation diameter of 8 mm (range 6–10). The median pre- and post-TIPS PSG were 17 mmHg (range 9–30) and 6 mmHg (range 0–12) respectively (*p* < 0.001). Patients’ and TIPS’ characteristics are detailed in Table 1.

**Table 1:** Characteristics of patients and TIPS procedures

Characteristics	Values
No	33
M/F	24 (72.7) / 9 (27.3)
Age (years)	56.8 (44–71)
BMI (kg/m2)	24.5 (14–48)
MELD score	11 (6–26)
Child–Pugh score	8 (5–14)
Renal failure	8 (24.2)
Diabetes	9 (27.3)
Concomitant cancer	4 (12.1)
Anticoagulation	10 (30.3)
β-blockers	13 (39.4)
History of variceal ligation or embolization	15 (45.5)
*Etiology of cirrhosis*	
Alcohol	20 (60.6)
Mixed	5 (15.2)
NAFLD	4 (12.1)
Viral	3 (9.1)
Hemochromatosis	1 (3.0)
*Indications for TIPS creation*	
Hemorrhage	16 (48.5)
Ascites	14 (42.4)
Prophylaxis	3 (9.1)
Stent-graft diameter (mm)	10 (8–12)
Tract dilatation diameter (mm)	8 (6–10)
Pre-TIPS PSG (mmHg)	17 (9–30)
Post-TIPS PSG (mmHg)	6 (0–12)

Values are given as n (%) or median (range)

*MELD* Model for End stage Liver Disease

*NAFLD* Non-Alcoholic Fatty Liver Disease

*BMI* Body mass index

*PSG* Portosystemic gradient

### TIPS revision

A total of 20 TIPS (60.6%) had revision within the first year after TIPS creation. The median time to TIPS revision was 10.4 months (range 0.2–86.8). TIPS revision’s characteristics are detailed in Table 2. Indication for TIPS revision was recurrent symptoms of portal hypertension in 13 (39.4%) patients, US dysfunction criteria alone in 12 (36.4%) patients, or both in 8 (24.2%) patients. Recurrent symptoms of portal hypertension were ascites in 15 (45.5%) patients, variceal hemorrhage in 4 (12.1%) patients, and worsening of gastric varices in 2 (6.1) patients. The pre-TIPS revision median PSG was 18.5 mmHg (range 8–28), higher than the post-TIPS PSG (*p* < 0.001).

**Table 2:** Characteristics of TIPS revision procedures

Characteristics	Values
No	33
*Indication for TIPS revision*	
Portal hypertension	13 (39.4)
US dysfunction criteria	12 (36.4)
Both	8 (24.2)
Pre-TIPS revision PSG (mmHg)	18.5 (8–28)
Post-TIPS revision PSG (mmHg)	8 (4–19)
*Proximal end TIPS location*	
Portal vein	23 (69.7)
Right portal vein branch	10 (10.3)
*Distal end TIPS location*	
HV	28 (84.8)
HV/IVC junction	5 (15.2)
*Patterns of TIPS DAF*	
1	23 (69.7)
2	5 (15.2)
3	2 (6.1)
4	3 (9.1)
*Stenosis rate*	
< 50%	5 (15.2)
> 50%	23 (69.7)
Complete occlusion	3 (9.1)
Missing	2 (6.1)
*Delay of TIPS revision*	
< 12 months	20 (60.6)
> 12 months	13 (39.4)
Successful TIPS revision	30 (90.9)

Values are given as n (%) or median (range)

*PSG* Portosystemic gradient

*HV* Hepatic vein

*IVC* Inferior vena cava

*TIPS* Transjugular intrahepatic portosystemic shunt

*DAF* Dysfunction-associated features

**Fig. 2:**
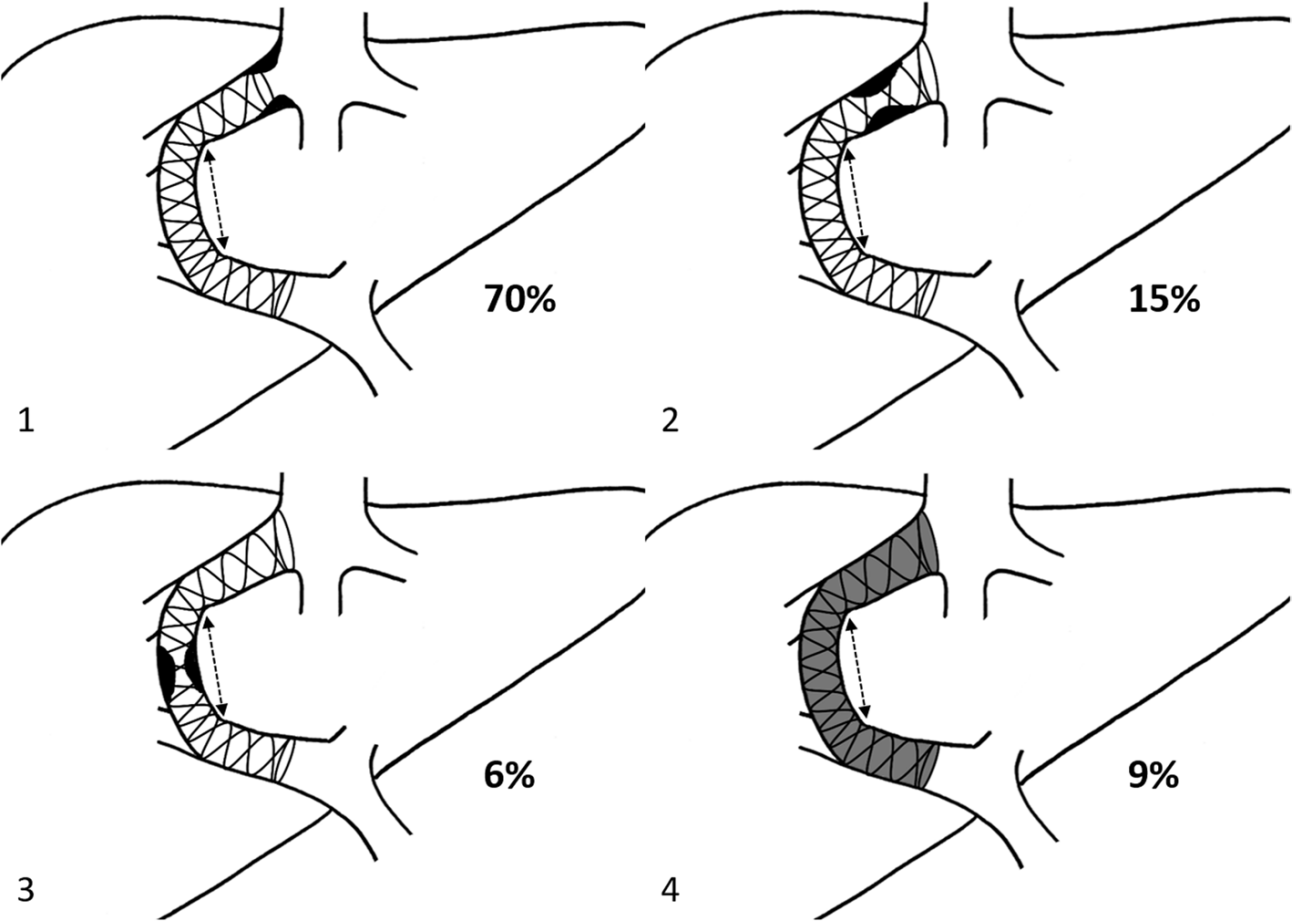
Patterns of TIPS dysfunction-associated features. Double-dotted arrows delineate the covered portion of the TIPS

On the angiogram, the proximal end of the TIPS was positioned within the main portal vein in 23 patients (69.7%) or the right branch of the portal vein in the other 10 patients (30.3%). The TIPS’ distal end was at the HV/IVC junction in 5 (15.2%) patients and within the HV in the remaining 28 (84.8%) patients. Angiograms showed four patterns of TIPS dysfunction-associated features (DAF) that were defined as follows (Fig. 2): Type 1 was defined as a stenosis located after the bare stent end in the HV. Type 2 was defined as stenosis within the bare stent in the HV. Type 3 was defined as intra-stent-graft stenosis in the parenchymal tract of the TIPS or a kink at the portal vein-parenchymal tract. Type 4 was defined as complete TIPS occlusion with no stenosis highlighted.

**Fig. 3:**
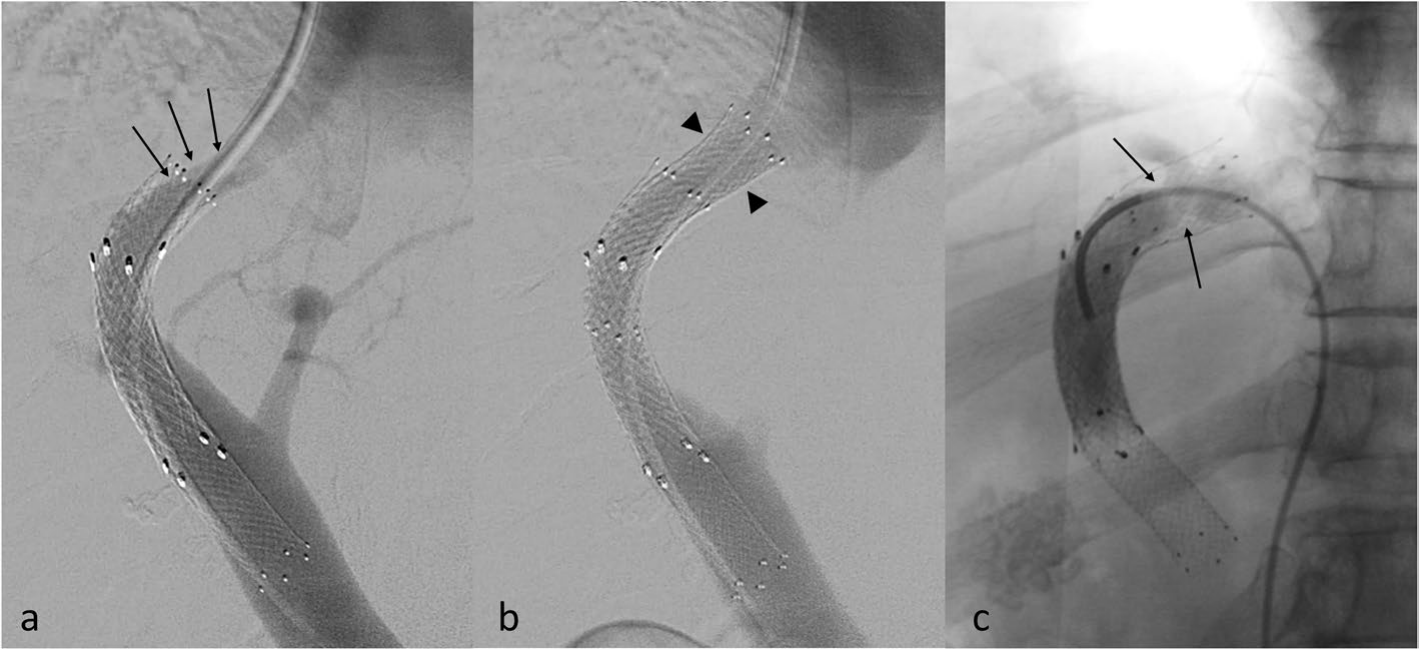
Types 1 and 2 TIPS’ dysfunction. **a** Angiogram showing a type 1 TIPS DAF with stenosis (arrows) located after the stent end in the hepatic vein. **b** Angiogram in the same patient after the addition of a new bare stent (arrowheads) up to the hepatic and inferior vena cava junction. **c** Angiogram showing a type 2 TIPS DAF with an intra-stent stenosis (arrows) located in the hepatic vein

At TIPS revision, types 1, 2, 3, and 4 of TIPS DAF were seen in 23 (69.7%), 5 (15.2%), 2 (6.1%), and 3 (9.1%) TIPS respectively. Examples of types 1 and 2 TIPS DAF are shown in Fig. 3.

On the angiogram, a total of 23 (69.7%) TIPS showed a stenosis rate above 50% while 5 (21.7%) showed a stenosis rate below 50%. Two (6.1%) stenoses were not measurable and 3 (9.1%) TIPS showed complete occlusion.

During TIPS revision, recanalization was performed in 30 patients (90.1%) and was successful in all cases with angioplasty alone in 5 patients (16.7%) and angioplasty and stents placement in 25 patients (83.3%). A total of 3 patients (9.9%) didn’t undergo angioplasty of the shunt because of hepatic encephalopathy. The median number of stents inserted at TIPS revision was 1 (range 1–3). The median stent diameter was 10 mm (range 10–14) with a median dilatation diameter of 10 mm (range 8–12). A total of 13 TIPS had secondary dysfunction with a median delay of 1.83 years (range 0.01–10.3) during a median follow-up of 3.13 years (range 0.05–15.1).

The median post-TIPS revision PSG was 8 mmHg (range 4–19), significantly lower than the pre-TIPS revision PSG (*p* < 0.001).

## Discussion

We use a generic stent-graft for the creation of TIPS in our center with the addition of bare metal stents in the hepatic and portal veins to leave the HV permeable. Many interventional radiologists use the Viatorr stent-graft, a nitinol self-expendable stent-graft with an uncovered segment of 2 cm in its proximal end designed to extend into the portal vein. The covered portion of the Viatorr stent-graft extending to the HV/IVC junction may be responsible for the occlusion of the targeted HV. Liver infarction is a rare complication of TIPS [3, 4, 22–24] and has been described since the use of bare metal stents, mostly related the arterial injuries [25–27]. Even though the impact of HV thrombosis on hepatic infarction occurrence remains unclear [7, 23, 28], covering the HV may increase the liver ischemia risk due to the obstruction of the venous outflow [8, 9] especially when associated with portal vein thrombosis [24], with the potential risk of liver failure and abscess formation [10, 11]. The incidence of HV thrombosis after TIPS creation using Viatorr sent-grafts has been reported in approximately 16% of patients [7].

TIPS creation using Fluency stent-grafts has shown good 1-year primary unassisted patency rates ranging from 81 to 93.1% [12–19]. Saad et al. and Wu et al. results suggest that TIPS creation using Viatorr stent-grafts provides higher primary patency rates [12, 15]. Reasons for a higher primary patency rate of the Viatorr in Saad et al. study [15] may be favored by inappropriate stent-graft placement in the Fluency group with stenoses occurring exclusively at the portal venous end caused by the proximal parenchymal tract being covered only by the bare stent. In Wu et al. study [12], a bare stent was not systematically associated with the Fluency stent-graft, and many patients were censored within the first year after TIPS creation in the Fluency group. We believe that the superiority of the Viatorr over the Fluency stent-graft should be confirmed in further studies.

TIPS dysfunction can be either the result of thrombosis or intimal hyperplasia, with the main cause being intimal hyperplasia [22]. Before the use of PTFE-covered stent-grafts, stenoses, and occlusions were mostly located within the hepatic parenchymal tract due to bile duct transection [29]. In our study, intimal hyperplasia of the HV was the main cause of TIPS dysfunction. Our results emphasize the usefulness of PTFE-covered stent grafts to cover the hepatic parenchymal tract, with stenoses being more likely to occur outside the hepatic parenchymal tract in the HV (types 1 and 2) in our series.

Suboptimal stent length seems to result in higher shunt dysfunction [30–32]. When the stent extends only in the HV and does not reach the IVC junction, the shunt flows and the resulting turbulence and shear stress could account for the acceleration of pseudo-intimal hyperplasia on the non-stented portion of the HV. Our results highlight the importance of appropriate stent length in the HV, consistent with prior results [30, 32], with approximately 70% of TIPS dysfunction in our study featuring a short stent in the HV with stenoses at the stent end (type 1). The low proportion of stenoses occurring inside the bare stent in the HV (type 2) suggests that covering the HV with PTFE-covered stent-grafts isn’t mandatory and that the HV could be left open when creating a TIPS.

The recommended target PSG after TIPS is 12 mmHg or less, or a reduction of at least 20% [2–4, 22, 33]. The post-TIPS PSG in our population was consistent with previous and current guidelines with all post-TIPS PSG being at 12 mmHg or below. The pre-TIPS revision PSG was significantly higher than the post-TIPS PSG and the post-TIPS revision PSG was significantly lower than the pre-TIPS revision PSG. However, TIPS revision procedures were performed either under general or local anesthesia while all TIPS placements were performed under general anesthesia when general anesthesia is known to lower PSG when compared to PSG measured in patients fully awake [34].

The major limitations of our study included the fact that it was a retrospective and single-center study. Also, 15 years elapsed between the first and the last TIPS creation. Over this period, many factors may have evolved, such as hardware, software, operators, and practices that may invoke some selection and outcomes bias. Finally, the shunt patency rate was not evaluated and a comparison with TIPS that did not require revision couldn't be performed as many patients were followed in other centers while all TIPS revisions of the area were referred to our center resulting in the fact that most patients followed in our center were the ones that had a shunt dysfunction. Of the 99 TIPS that were created between January 2005 to December 2020, 20 had a TIPS revision within the first 12 months, consistent with the expected 15–20% shunt dysfunction rate at 1 year [2, 33] and previous studies [12–19].

In conclusion, our results illustrate the four angiographic patterns of shunt DAF after TIPS creation using a combination of a generic stent-graft and bare-stents. Further studies are warranted to confirm our results.

## Data Availability

The datasets used and/or analyzed during the current study are available from the corresponding author on reasonable request.
